# The divergence between T cell and innate lymphoid cell fates controlled by E and Id proteins

**DOI:** 10.3389/fimmu.2022.960444

**Published:** 2022-08-10

**Authors:** Aneta Pankow, Xiao-Hong Sun

**Affiliations:** ^1^ Program in Arthritis and Clinical Immunology, Oklahoma Medical Research Foundation, Oklahoma City, OK, United States; ^2^ Department of Microbiology and Immunology, University of Oklahoma Health Sciences Center, Oklahoma City, OK, United States; ^3^ Department of Cell Biology, University of Oklahoma Health Sciences Center, Oklahoma City, OK, United States

**Keywords:** E2A, HEB, innate lymphoid cells, Id2, Id3, T cells

## Abstract

T cells develop in the thymus from lymphoid primed multipotent progenitors or common lymphoid progenitors into αβ and γδ subsets. The basic helix-loop-helix transcription factors, E proteins, play pivotal roles at multiple stages from T cell commitment to maturation. Inhibitors of E proteins, Id2 and Id3, also regulate T cell development while promoting ILC differentiation. Recent findings suggest that the thymus can also produce innate lymphoid cells (ILCs). In this review, we present current findings that suggest the balance between E and Id proteins is likely to be critical for controlling the bifurcation of T cell and ILC fates at early stages of T cell development.

## Introduction

**Figure 1:**
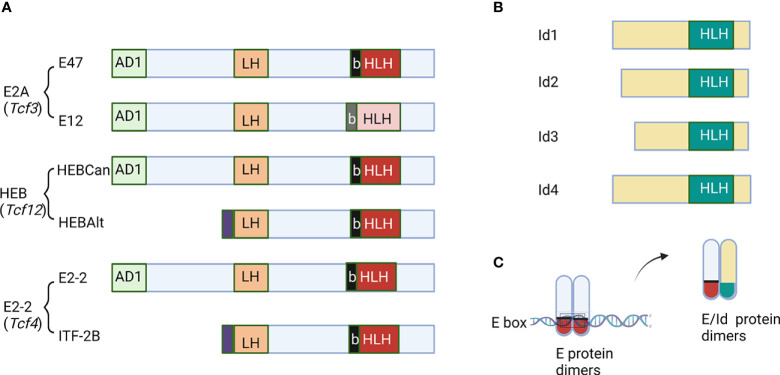
Schematic diagrams of E and Id proteins. **(A)** The function domains of E proteins are labeled. AD1 and LH are two transcription activation domains. The basic and helix-loop-helix domains are marked as b and HLH, respectively. **(B)** Id proteins with the HLH domain are shown. **(C)** The mechanism of action of Id proteins to inhibit DNA binding by E proteins. The figure was created by BioRender.com.

The E protein family of transcription factors are crucial molecules engaging in B cell development in the bone marrow and T cells differentiation in the thymus (1, 2). This family consists of proteins encoded by three genes, E2A (also called *Tcf3*), HEB (*Tcf12*) and E2-2 (*Tcf4*) (**Figure 1A**) (3–5). These proteins share extensive sequence homologies in the activation domains (AD1, LH) and basic helix-loop-helix (bHLH) DNA-binding domain (6–9). E proteins regulate the transcription of their target genes by forming homodimers or heterodimers and bind to E-box sequences (9). The E2A gene gives rise to two proteins, E12 and E47, due to alternative splicing of two adjacent exons, each encoding a basic helix-loop-helix (bHLH) domain (10). While E47 binds DNA avidly as homodimers, E12 does so poorly due to the presence of an inhibitory domain (11). However, both form heterodimers with other bHLH proteins such as MyoD, and bind DNA efficiently. The HEB gene encodes a full-length canonical protein (HEBCan) and a truncated alternate form (HEBAlt), which derives from a transcript initiated in the middle of the gene (12). HEBAlt lacks the AD1 transcription activation domain and has lower transcriptional activities (13). It has an Alt domain at the N-terminus with three tyrosine residues which can be modified by phosphorylation that augments its transcriptional activity (13).

The family of inhibitor of differentiation proteins, Id1-4, antagonize E proteins by dimerizing with them *via* the helix-loop-helix domain (**Figure 1B**) (14–19). However, because Id proteins lack the basic amino acids necessary for DNA binding, heterodimers between E and Id proteins cannot bind to E box sequences (**Figure 1C**). Transcription of the E protein genes is less variable but that of the Id genes is highly dynamic. Therefore, the net E protein activity in a given cell is determined by the levels of both E and Id proteins (16, 17). In this review, we intend to highlight the roles of E and Id proteins in regulating the fate choices between T cells and innate lymphoid cells.

## T cell development

Lymphoid-primed multipotent progenitors (LMPP) and common lymphoid progenitors (CLP) travel from the bone marrow to the thymus and become early T cell progenitors (ETP) (20–23). T cell developmental progression in the thymus can be generally defined by the expression of CD4 and CD8 surface markers: from CD4 and CD8 double negative (DN) to double positive (DP) and then to CD4 or CD8 single positive (SP) (24–27). During the transition from DN to DP stage, an immature CD8 single positive subset (ISP) has been described (28, 29). Within the DN compartment, four subsets (DN1 to DN4) are characterized by the expression of c-kit and CD25 in the order of maturity as c-kit^+^CD25^-^, c-kit^+^CD25^+^, c-kit^-^CD25^+^ and c-kit^-^CD25^-^ (27). ETPs are at the top of the hierarchy and included in the DN1 subset (23). They give rise to both αβ and γδ T cells, which have distinct T cell receptors (TCRs), different developmental programs and divergent functions. The E2A and HEB genes are both expressed in the thymus. Interestingly, HEBAlt is preferentially produced in the DN and ISP stages. Since E proteins are known to inhibit cell proliferation and HEBAlt acts as a hypomorph (13, 30), whether HEBAlt plays a role in tampering E protein activities during pre-TCR-triggered cell expansion is interesting to be investigated.

### αβ T cells

The development of αβ T cells is largely driven by αβ TCR signaling events. However, before the formation of pre-TCRs and TCRs, the differentiation of committed T cell precursors is supported by Notch signaling and signaling from cytokine receptors such as that of IL-7 (31–35). Critical transcription factors involved in T cell commitment include TCF1, GATA3 and Bcl11b (36–40). The sequential rearrangements of TCRβ and then TCRα genes catalyzed by the RAG1 and RAG2 recombinases set the milestones of the developmental progression (24, 41–44). The TCRβ locus undergoes recombination between D to J regions and then V to DJ regions to produce functional β chains, which pair with the pre-Tα (45, 46). The pre-TCR complex delivers signals leading to the expansion of DN3 cells and their advancement to the DP stage. The TCRα gene rearrangement occurs at the DP stage, which allows the formation of αβ TCRs, triggers the positive and negative selection and enables the generation of mature SP T cells (47–49). Mature but naive T cells leave the thymus by the upregulation of S1PR1 and CD62L (50–53).

αβ T cells possess a large repertoire of TCRs due to a collection of V regions. These TCRs recognize diverse antigens presented by the MHC molecules and elicit subsequent signaling events. CD4^+^ and CD8^+^ naïve T cells exit the thymus to be activated and differentiate into helper and cytotoxic effectors in peripheral lymphoid organs, respectively (54, 55). Due to the sheer quantity of thymic output of αβ T cells and their ability to proliferate in response to antigen engagement, αβ T cells are the major players of adaptive T cell immunity.

### γδ T cells

The development of γδ T cells differs from αβ T cells. Firstly, unlike αβ T cells, γδ T cells do not traverse DP and SP stages during the development. Instead, they undergo γδ lineage commitment and maturation at DN2 and DN3 stages (56–58). Generation of mature γδ T cells depends on the V-J rearrangement of the TCRγ locus and V(D)J recombination in the TCRδ locus, along with Notch signaling. Since the TCRδ gene is embedded in the TCRα locus, TCRα rearrangement, triggered by pre-TCR signaling after an independent rearrangement event of the TCRβ gene, can eliminate the TCRδ gene, thus aborting the γδ T cell fate (59–61). Early precursors of effector γδ T cells in the thymus are identified as CD24^+^ and then mature to CD24^-^ stage (62, 63). There are three types of γδ T cells classified based on their effector functions, γδ1, γδ17 and innate-like γδ T cells, which secrete interferon γ, IL-17 and interferon γ plus IL-4, respectively (64–66). The development of γδ T cells require stronger TCR signals in the comparison to αβ T cells (67, 68). The gradients of TCR signals determines the development of specific effector subsets. The generation of innate-like γδ T cells depends on the strongest TCR signal as indicated by their higher levels of CD5 compared to other γδ subsets (69). CD5 levels are proportional to TCR signaling strength in the thymus (70, 71). Expression of PLZF transcription factor also depends on ligand ligation with TCR and PLZF is required for the effector function of innate-like γδ T cells (72). Type 1 γδ T cells also require a strong TCR signal and the T-bet transcription factor is critical for γδ1 differentiation (65, 72–75). On the other hand, type 17 γδ T cells rely on a weaker TCR signal for the differentiation (65, 72, 74, 76). In fetal organ culture, addition of activating antibodies against γδ TCR or CD3 impairs the production of γδ17 cells (76). Moreover, RORγt transcription factor is essential for γδ17 development (77). Additionally, CD73 expression marks most of γδ T cells committed to mature into effector cells in the thymus (78).

Distinct subsets of γδ T cells reside in different tissues and develop at different ages in mice (56, 57). The Vγ regions are described by two different nomenclatures. In this review, we will use the one defined by Raulet and colleagues (79). In the early fetal stage, the first wave of γδ T cells is associated with the Vγ3^+^Vδ1^+^ subset known as the dendritic epidermal cells, which produce IFNγ (65, 80, 81). The development of the Vγ4^+^ subset begins at the fetal stage and lasts until birth. The generation of the Vγ2^+^ subset occurs in the late fetal stage and continues through adulthood. The Vγ2^+^ subset consists of cells producing IL-17 or IFNγ (82). The IL-17-producing cells become long-lived cells with self-renewal capabilities after birth (83). Vγ1.1^+^ cells develop at the prenatal stage and this persists through adult life (56). Despite the complicated developmental schemes of γδ T cell differentiation, how γδ TCRs interact with their ligands and elicit signals is less understood. To some extent, γδ T cells are thought to have properties resembling innate cells.

T cell development is a “wasteful’ process”. Every D-J or V-DJ combination only has one third of a chance to create an in-frame joint that result in a full-length TCR chain. It is believed that over 70% of the developing T cells do not reach the mature stage and die because they fail to form pre-TCR (β selection) at the DN3 stage or because they cannot produce a full-length TCRα chain at the DP stage (death by neglect). They can also be eliminated due to excessively strong TCR signaling (negative selection). Are there any alternative fates for these T cell “drop-outs”? Perhaps, innate lymphoid cells are some of the options.

## Regulation of T cell development by E and Id proteins

E proteins play pivotal roles in governing the development of αβ T cells. Two of the E protein genes, E2A and EBCan, are expressed in T cells and they have redundant functions. The proteins encoded by these two genes include E12, E47, HEBCan and HEBAlt. Since all knock-out constructs targeted the bHLH domains, E2A or HEB deficient mice lack all of their respective proteins. Germ-line ablation of either E2A or HEB gene partially impairs T cell development by dramatically reducing thymocyte counts (84, 85). The leaky block allows the maturation of small numbers of T cells, which are predisposed to develop T cell lymphoma (84–86). HEB deficiency also reveals a novel role of HEB at the ISP stage (86). In contrast, simultaneous inhibition of all E proteins by expressing Id1 using the proximal promoter of *lck* in transgenic mice results in a complete block of T cell development, arresting thymocytes at the DN1 stage when the Id1 transgene begins to be expressed (87, 88). Likewise, inducible ablation of both E2A and HEB genes using the *plck-Cre* transgene results in a developmental arrest at the DN3 stage when the Cre gene is expressed (89).

E protein-mediated control at these early stages of T cell development is multi-dimensional. First, E proteins are known to activate the transcription of *Notch1*, which encodes the receptor for Notch ligands such as Delta-like 4 in the thymus and ensure the differentiation and survival of T cells (90–92). Second, E proteins are found to activate the transcription of *Rag1* and *Rag2* (93, 94), which code for the enzymes essential for VDJ recombination of TCR genes. Third, E proteins facilitate TCR gene rearrangement by increasing chromatin accessibility at the TCRβ locus (95). Fourth, the binding of E2A-HEB heterodimers to *Ptcra* enhancer regulates pre-Tα expression at the DN3 stage (96–98). Finally, the interplay between E proteins and other transcription factors such as TCF1 and LEF1 also contribute to the positive regulation of early T cell development (36, 99).

Following pre-TCR signaling, the Ras-MAP kinase pathway is activated, which leads to the up-regulation of Egr transcription factors and then activation of the Id3 gene (100–103). This suggests that down-regulation of E protein activity is necessary for DN3 cells progress to the DP stage. Indeed, when *Rag1* was deleted, T cell development arrested at the DN3 stage (104). However, if E proteins are down-regulated by germline E2A deletion or *pLck-Id1* expression, *Rag1* ^-/-^ thymocytes can advance to the DP stage (105, 106). Another mechanism to down-regulate E proteins is to accelerate their ubiquitin-mediated degradation in the presence of Notch signals and MAP kinases activated by pre-TCR signaling (107, 108).

At the DP stage, Id3 expression is transiently triggered by TCR signaling and is involved in the positive selection of developing thymocytes (101, 109). Deleting both Id2 and Id3 genes prevented the progression of positively selected T cells to the SP stage (110). Conversely, low levels of Id1 expression in *plck-Id1* heterozygous transgenic mice allows some T cell precursors reach the DP stage but a majority of these cells undergo apoptosis likely due to excessive responses to the normal levels of TCR stimulation (105, 111). This notion was supported by the observation of hyper-activation of NFκB upon ectopic Id1 expression (105, 112). In addition, deleting both E2A and HEB genes also impairs the generation of CD4 SP T cells (110). Collectively, E and Id proteins clearly are the central players in shaping αβ T cell development.

A strong TCR signal triggers the activation of the ERK-Egr-Id3 axis and favors γδ over αβ T cell development (73). Id3 deficiency resulted in an expansion of Vγ1.1^+^ innate-like γδ T cells, possibly due to the dampening of the strong TCR signaling which normally causes the death of these cells (113, 114). In fetal organ cultures, HEB deficiency impairs the differentiation of Vγ4 and Vγ6-containing γδ17 cells. In *et al.* postulated two pathways of γδ T cell development (115). Pathway 1, which favors γδ1 cells, depends on strong TCR signaling and up-regulation of Id3. In contrast, pathway 2 mostly occurs in the fetal stage and requires lower levels of TCR signaling and Id3 expression. HEB is necessary for Vγ6^+^CD73^-^ γδ17 T cells in the fetal stage as well as Vγ4^+^CD73^+^ γδ17 T cells in neonates (115). HEB and E2A are thought to activate the transcription of *Sox4, Sox13* and *Rorc* genes necessary for γδ17 differentiation (115, 116). Overall, it appears that Id3 expression plays a critical role in directing γδ T cell development through counterbalancing the function of E proteins.

## Differentiation of innate lymphoid cells

Innate lymphoid cells (ILCs) are first responders in immune reactions towards environmental insults and microbial infections. ILCs are divided into three groups, ILC1 to ILC3, which play different roles during specific immune responses (117, 118). Even though ILCs share with T cells the transcriptional factors that drive their differentiation and the profiles of cytokine production, they lack T-cell receptors (TCR), thus eliciting innate immunity as opposed to adaptive immunity mediated by T cells (118–120). Each ILC subset has been increasingly recognized to be heterogenous and display different characteristics in different tissues (121). Plasticity between the three ILC subsets also exist, especially under pathophysiological conditions (118, 122). Nevertheless, the general properties and functions of these three subsets of ILCs have been established. The ILC1 group consists of helper-like ILC1s and conventional NK cells (cNK). ILC1s mediate the early immune response upon contact with intracellular pathogens like bacteria and viruses. Their effector function regarding cytokine production is similar to the that of cNK cells, namely secreting IFNγ upon pathogen exposure. However, NK cells but not helper-like ILC1s are cytotoxic and able to produce high levels of cytotoxic granules like perforin and granzymes. The T-bet transcription factor is responsible for ILC1 differentiation and function (123). ILC2s share a transcriptional network and cytokine production profiles with those of type 2 T helper cells (Th2). GATA3 is the signature transcription factor and drives the expression of cytokines including IL-5, IL-13, IL-4, IL-9, and amphiregulin (124–126). RORα is another transcription factor indispensable for ILC2 differentiation (127). ILC2s are crucial for the protection against helminth infection. They are also activated by allergens due to the release of IL-25, IL-33 and TSLP in the tissues, contributing to a number of respiratory diseases such as asthma (128). On the other hand, ILC2s have also been shown to be involved in tissue repair following influenza infection (129). The ILC3 group includes innate immune cells committed to targeting extracellular microbes. They reside mainly in the mucosal tissues and maintain their homeostasis locally. ILC3s express RORγt and produce cytokines such as IL-17A, IL-22, and GM-CSF (118, 123). Lymphoid tissue inducers (LTis) are a subset of ILC3s essential during the fetal stage for supporting the development of lymph nodes and other lymphoid tissues (130).

Innate lymphoid cells are progenies of hematopoietic stem cells, arising from progenitors destined to become lymphoid cells such as lymphoid-primed multipotent progenitors (LMPPs) or common lymphoid progenitors (CLPs) (20, 21). These progenitors reside in fetal liver or adult bone marrow where ILCs differentiate in addition to B cells. These processes have been extensively studied as summarized below. However, LMPPs and CLPs also travel to the thymus to produce T cells. The capability of the thymus to support ILC differentiation has recently become appreciated (90, 131–133). The divergence of T cell development to ILC fates is an interesting issue to be addressed here. Finally, ILCs are also believed to be derived from tissue-resident progenitors but at what stage these progenitors seed the peripheral tissues and whether all ILC subsets utilizes this mechanism of reproduction are not fully understood.

### ILC differentiation in the bone marrow and fetal liver

Innate lymphoid cells develop in the bone marrow from LMPPs or CLPs through a series of intermediate progenitors which progressively lose the potential of giving rise to B cells and then NK cells (120). The progenitors that can generate subsequent progenitors for either ILC or NK cells are called alpha LPs (αLPs), which require the NFIL3 and TOX transcription factors (134). Early innate lymphoid progenitors (EILP) characterized by TCF1 expression, also have a similar differentiation potential (135). Next, common helper ILC progenitors (CHILPs) are regulated by Id2 and responsible for the ILC but not NK subsets (136). ILC progenitors (ILCPs) controlled by PLZF are dedicated to only producing ILCs, and are found in both bone marrow and fetal liver (137). In contrast, NK progenitors (NKPs) which also express Id2 are specialized to become NK cells (120, 137). Although CHILPs or ILCPs have the potential to give rise to all three ILC subsets *in vitro* when cultured on OP9-DL1 stroma, the predominant subset detected in the bone marrow is ILC2 as well as their precursors called ILC2Ps (138). Moreover, there is also evidence that ILC1s can be generated in adult liver from fetal hematopoietic stem cells (139).

Whether the bone marrow serves as a constant source of ILC2 replenishment has not been well established. Experiments using parabionts suggested tissues such as the lung receive few ILC2s from the blood circulation (140, 141). However, recent single cell RNA sequencing (scRNAseq) data showed a population of ILC2s in the blood of wild type and athymic nude mice, which suggest that these ILC2s may come from the bone marrow or they are the recirculating ILC2s from peripheral tissues (133). IL-18R^+^ precursors of ILC2s have also been found in the lung and shown to arrive from the blood (142, 143).

In humans, ILC progenitors with biases to different ILC subsets are readily detectable in the blood (144, 145). Likewise, committed ILC1 to ILC3 subsets are also found in the blood (122, 146). These cells are assumed to come from the bone marrow but no direct evidence is available. The frequencies of the ILC subsets are often found to be altered in different disease states, which may potentially serve as biomarkers of these diseases (147–149).

### ILC differentiation in the thymus

Small numbers of ILCs, particularly ILC2s, have been found in the thymus at pre- and post-natal stages (150–154). This is consistent with the fact T cell progenitors express the transcription factors supporting ILC2 differentiation, namely GATA3, TCF1 and Bcl11b (155).

Whether the thymus is another lymphoid organ capable of exporting ILC precursors or ILCs to peripheral tissues was investigated by using scRNAseq of the lineage negative (Lin^-^) Thy1^+^ fraction of the blood of wild type and athymic nude mice (133). Bajana *et al.* found that about half of the ILC-containing Lin^-^Thy1^+^ population, was greatly diminished in the athymic nude mice, which suggest that the production of these cells is thymus-dependent, thus designated td-ILCs. These cells were fractionated into four clusters based on their distinct transcriptomic properties. All td-ILCs express genes commonly expressed in ILCs such as *Tcf7* and *Il7r* but they lack the signature transcription factors that specify ILC1 to ILC3: T-bet, GATA3 and RORγt, suggesting that td-ILCs can be ILC precursors. Indeed, when these cells were isolated as Lin^-^Thy1^+^CD127^+^CD62L^+^ from the blood and cultured on OP9-DL1 stroma, different subsets of ILCs were generated (133). Whether this population contains disparate progenitors for distinct ILC subsets or progenitors with multiple potentials is to be determined.

Interestingly, td-ILCs express *Cd3d*, *Cd3e* and *Cd3g* but no other T cell specific genes such as *Cd4, Cd8a, Rag1, Rag2* and *Dntt*. Flow cytometry analyses detected CD3ϵ by intracellular staining but not by surface staining (133). Moreover, td-ILCs do not have TCRβ or TCRδ either on the surface or in the cytoplasm, thus indicating that they are not T cells. Using intracellular CD3ϵ (icCD3ϵ) as a marker, Bajana *et al.* also detected icCD3ϵ^+^ cells in the lung, small intestine and skin of wild type mice (133). Because these icCD3ϵ ^+^ cells are greatly diminished in nude mice, the results were interpreted to mean that icCD3ϵ marks thymus-derived cells. Like in blood td-ILCs, the icCD3ϵ^+^ cells in the lung and small intestine do not express TCRβ or TCRδ, ruling out the possibility that they are T cells. This suggests that td-ILCs in the blood may home to peripheral tissues where they differentiate into diverse ILC subsets. In the lung, a significant fraction of icCD3ϵ^+^ ILCs are ST2^-^RORγt^+^ ILC3-like cells. In contrast, the lamina propria of small intestine harbors icCD3ϵ^+^KLRG1^-^T-bet^+^ ILC1-like cells. Curiously, the expression levels of GATA3 correlated inversely with those of icCD3ϵ, which suggests that ILC2 differentiation is accompanied by the down-regulation of CD3 expression (133). Although this possibility remains to be investigated, the potential down-regulation of CD3 expression makes it difficult to assess the contribution of thymus-derived ILC2s to the overall ILC2 pool. A lineage-tracing system with a *Cre* transgene that is specifically and efficiently expressed at the early stages of T cell development would greatly facilitate the estimation of the contribution of thymus-derived ILC2s and further validate the thymic origin of ILC2 subsets.

Additional evidence exist that support the notion that the thymus contributes to the ILC2 pools. Qian *et al.* showed that not only multipotent progenitors (DN1) but also committed T lineage cells (DN3) from the thymus can differentiate into functional ILC2 on OP9-DL1 stromal cells (132). Consistently, ILC2s isolated from the lung of WT but not nude mice harbor rearranged TCR genes, *Tcrb* and *Tcrg*, suggesting that at least some of the ILC2s originated from committed T lineage cells in the thymus (132, 156). While *Tcrg* rearrangement was readily detectable by electrophoresis, analyses of the D-J and V-DJ recombination in the *Tcrb* locus required Southern blotting because of the diversity of their rearrangement events. Shin *et al.* sequenced the rearranged *Tcrg* segments and found a reduced frequency of in-frame rearrangement in ILC2s compared to that in γδ T cells (156). It was thus concluded that ILC2s are derived from cells which have failed productive γδ TCR rearrangement (156, 157). However, further investigation at the single-cell level could strengthen the conclusion. Despite the rearrangement events detected, ILC2s do not express TCRβ or TCRδ either intracellularly or on the surface.

Likewise, NK cells have also been shown to arise from early T cell precursors in the thymus, suggesting a branch point between T and NK cells (158–160). It remains to be determined if this branch point is similar or different from those giving rise to ILCs.

## Regulation of ILC differentiation by E and Id proteins

*Id2* is expressed in ILC progenitors and plays an essential role in ILC development, which implicates the involvement of E proteins in regulating ILC differentiation (136, 161). Strikingly, down-regulation of E proteins by the ectopic expression of Id1 in transgenic thymocytes at the DN1 stage or by deletion of the E2A and HEB genes with *plck-Cre* at the DN3 stage led to dramatic increases in ILC2 production in the thymus (131, 132). As a result, large amounts of ILC2s were exported from the thymus to peripheral tissues throughout the body. The thymus was shown to be responsible for the mass production of ILC2 in Id1 transgenic mice because when the transgene was bred onto the nude background, ILC2 expansion was no longer detectable (132). ILC2s made in the thymus of Id1 transgenic and E protein deficient mice respond to IL-25 or IL-33 stimulation similarly as wild type ILC2s by secreting IL-5 and IL-13 in cultures (131, 132). *In vivo*, Id1 transgenic mice exhibited greater type 2 responses when treated with papain in the lung or during helminth infection (131). These are likely due to the presence of excessive amounts of ILC2s in Id1 transgenic mice. However, on a per cell basis, Id1 transgenic ILC2s appeared to have a less robust production of IL5 and IL-13 (131). It is not clear if this is due to a cell intrinsic difference or a limitation of stimuli available to all of the extra ILC2s in Id1 transgenic mice. Barshad *et al.* made a similar observation by treating wild type and Id1 transgenic mice with house dust mites (HDM) (162). By analyzing the chromatin accessibility, they found a reduction in AP-1 and C/EBP binding sites in open chromatins after HDM treatment in Id1 transgenic ILC2s. Whether this is due to a direct or indirect effect of E protein inhibition remained to be determined.

In the blood of *Tcf3^fl/fl^Tcf12^fl/fl^plck-Cre* mice, an extremely large population of cells (cluster 0) that belong to thymus-dependent ILC precursors was detected using scRNAseq (133). In addition, a subset (cluster 2) with characteristics of NK cells was also markedly enriched (133). These cells can give rise to different ILC/NK subsets when cultured on OP9-DL1 stroma (133). Together, these results suggest that E proteins play multiple roles in suppressing the production of ILC and NK precursors, which may arise at different developmental stages or from different T cell precursors. Whether E proteins suppress the same or different transcriptional programs governing ILC and NK differentiation remains to be investigated.

Ablating E2A and HEB genes starting at the CLP stage using *IL7r-Cre* increased the production of both ILC2s and LTi-like cells, a subset of ILC3s (90). Conversely, inducible expression of a gain-of-function mutant of E47 by *Rag1-Cre* impaired the differentiation of ILC2s from ILCP in the bone marrow (163). Furthermore, Id2^-/-^ mice have been shown to be devoid of NK cells and lymph nodes which are initiated by LTi cells (130). Yet, overexpression of Id3 in human hematopoietic stem cells promoted NK differentiation (164). These findings suggest that down-regulating E protein function is instrumental for NK cell differentiation (165). It was further shown that Id2 plays a key role in regulating the production of IL-15 important for NK homeostasis (166, 167).

## Transcriptional programs of E protein-mediated suppression of ILC differentiation

Inducible deletion of the E2A and HEB genes promoted ILC2 differentiation from CLP, DN1 and DN3 cells on OP9-DL1 stroma by 20-40 folds, which demonstrates a powerful cell-intrinsic suppression by E proteins (132). It is therefore interesting to elucidate the transcriptional programs that underlie the suppression of ILC2 differentiation. Miyazaki *et al.* performed RNA sequencing and Assay for Transposase-accessible Chromatin Sequencing (ATAC-seq) using DN1 (ETP) cells of fetal thymi of control and *Tcf3^fl/fl^Tcf12^fl/fl^Il7r*-*Cre* mice. As expected, they found the down-regulation of an array of genes important for T cell development, which include *Notch1, Ptcra, Rag1, Rag2* and *Cd3d*. On the other hand, genes known to be expressed in ILC progenitors or ILC2s were up-regulated. Examples of such genes are *Pdcd1, Il18r, Id2, Gata3, Lmo4, Rora, Tox, Est1, Il4, Il1rl1* and *Klrg1.* The chromatin accessibility assays also showed a shift from the open chromatin patterns of T cells to those of ILCs. While these findings agree with the phenotypes of E protein deficient mice, it is difficult to pinpoint the critical switches that alter the cell fates.

Likewise, Qian *et al.* conducted RNA sequencing using DN1 or DN3 cells from control and *Tcf3^fl/fl^Tcf12^fl/fl^Rosa26^CreERT2^* mice cultured on OP9-DL1 stromal cells (132). On day 4 of the culture, tamoxifen was added to the medium and the cells were collected 24 or 72 hours later. Expression of genes important for T cell development decreased whereas those crucial for ILC2 differentiation increased. Even after one day of E-protein ablation, a collection of genes coding for diverse transcription factors became activated. These include *Zbtb16, Gata3, Rora, Rxra, Klf6, Ikzf2* and *Irf4*. While it is possible that E proteins individually repress the transcription of all of these genes, a coordinated program that controls the transcription of critical factors essential for ILC2 differentiation may be at play.

A close-up look at the action of E proteins was carried out by making use of the E47-ER fusion proteins (112), which allowed instant induction of E protein activity upon addition of tamoxifen (168). ILC2s from the thymus of Id1 transgenic mice were transduced with retroviruses expressing E47-ER or empty control viruses. Transduced cells were isolated by sorting for EGFP expressed off the same retroviral vector. After expansion, these cells were then incubated with tamoxifen for 4 or 16 hours and harvested for RNA sequencing or ATAC-seq. Consistent with the function of E proteins as transcription activators, Peng *et al.* found more genes activated than repressed by E47-ER at both time points (168). Among them are three genes encoding transcriptional repressors, *Cbfa2t3, Jdp2 and Bach2* (169–171).

Interestingly, ATAC-seq data showed that a modest increase in chromatin accessibility 4 hours post induction of E47 was followed by a widespread reduction in open chromatin regions 16 hours later. Moreover, the transcription factor motifs enriched in the differential peaks shifted from those bound by bHLH and Ets1 proteins at 4 hours to those recognized by bZip and GATA factors. It is therefore possible that one of the mechanisms whereby E proteins suppress ILC2 differentiation is to control the expression of transcription repressors, which in turn negatively regulate the transcription of genes important for ILC2 differentiation or function. Although this hypothesis has not been validated through genetic complementation studies, the correlation between the alteration of gene expression in *Cbfa2t3* ^-/-^ and E protein deficient mice support this idea (168, 172). Proteins bound to bZip and GATA motifs such as Batf and GATA3 are also known to be crucial for ILC2 function (126, 173, 174).

The RORα transcription factor also plays an important role in ILC2 differentiation (127). *Rora* ^-/-^ mice lack ILC2s but have intact T cell compartments. Recently, Ferreira *et al.* showed that RORα promotes ILC2 over T cell development by activating the transcription of *Id2* and *Nfil3*, which in turn inhibit the function of E proteins (153). However, in E protein deficient thymocytes, *Rora* expression is consistently up-regulated (90, 132, 168). Thus, a positive feedback loop may perpetually cause the up-regulation of *Rora* expression during ILC2 differentiation. There are likely additional transcription factors which act in parallel or in sequence to coordinate the differentiation of ILC2s and possibly ILC progenitors. However, it is clear that E proteins and their inhibitors, Id proteins, play a central role in maintaining the balance between T cell and ILC development.

## The crossroads of T cells and innate lymphoid cells

**Figure 2:**
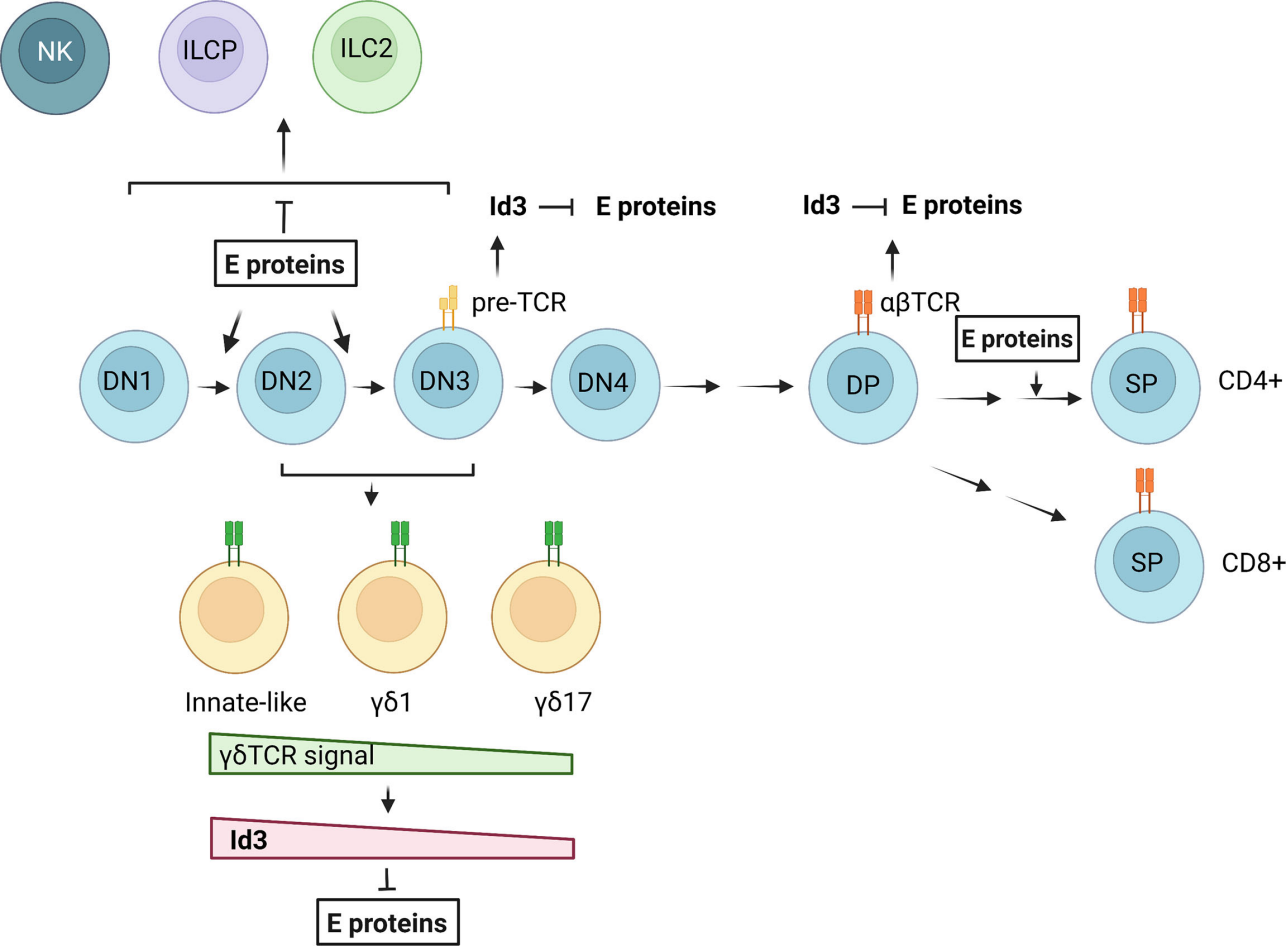
Regulation of T cell and ILC differentiation by E and Id proteins. E proteins promote T cell commitment and differentiation from DN1 to DN3 stages. Pre-TCR and TCR signaling in αβ T cells lead to transient Id3 up-regulation and E protein inhibition. In γδ T cell development, a gradient of γδ TCR signal determines the outcomes of different γδ subsets through regulation of Id3 expression and E protein activities. NKs, ILC2s and ILC precursors (ILCPs) may arise in the DN stages when E protein functions are suppressed. The figure was created by BioRender.com.

The major difference between T cells and innate lymphoid cells is the presence and absence of TCRs on their cell surface, respectively. However, there are a number of common features in the differentiation of these two types of cells (175). The thymic environment is conducive to the maturation of both T cells and ILCs (at least ILC2s and ILC3s) by supporting Notch and IL-7 signaling. The thymic progenitors equipped with transcription factors such as TCF1 and GATA3, are able to differentiate into both T cells and ILCs. Obviously, T cell production is the dominating responsibility of the thymus. This is due to the overwhelming effects of TCR-driven T cell expansion and powerful transcriptional programs in place to ensure an adequate T cell output. One of such transcriptional programs is controlled by the balance between E and Id proteins (**Figure 2**). When E protein activities are high, T cell development proceeds. When Id proteins overcome E proteins, ILCs can develop. Although Id2 has been shown to be expressed in ILC progenitors and play critical roles in ILC differentiation in the bone marrow, expression of Id3 is stimulated by TCR signaling in both αβ and γδ T cells (73, 106). This would create opportunities for developing T cells to divert to the ILC path. However, this possibility needs to be vigorously investigated. It is also interesting to explore whether the large numbers of developing T cells eliminated during the differentiation processes could be recycled into ILCs and used to replenish ILC pools in peripheral tissues. The E/Id axis has clearly been shown to be gate-keepers in the crossroads to T cell and ILC fates but the downstream transcriptional events remain to be further elucidated as the technologies and critical reagents become available.

## Author contributions

AP and X-HS wrote the manuscript. All authors contributed to the article and approved the submitted version.

## Funding

This work and funds for publication are supported by the National Institute of Allergy and Infectious Diseases (R56A126851).

## Conflict of interest

The authors declare that the research was conducted in the absence of any commercial or financial relationships that could be construed as a potential conflict of interest.

## Publisher’s note

All claims expressed in this article are solely those of the authors and do not necessarily represent those of their affiliated organizations, or those of the publisher, the editors and the reviewers. Any product that may be evaluated in this article, or claim that may be made by its manufacturer, is not guaranteed or endorsed by the publisher.

## References

[1] Murre C. Helix-loop-helix proteins and lymphocyte development. Nat Immunol (2005) 6(11):1079–86. doi: 10.1038/ni1260 16239924

[2] Lazorchak A Jones ME Zhuang Y. New insights into e-protein function in lymphocyte development. Trends Immunol (2005) 26(6):334–8. doi: 10.1016/j.it.2005.03.011 15922950

[3] Murre C McCaw PS Baltimore D. A new DNA binding and dimerization motif in immunoglobin enhancer binding, *daughterless*, MyoD and *myc* proteins. Cell (1989) 56:777–83. doi: 10.1016/0092-8674(89)90682-X 2493990

[4] Hu JS Olson EN Kingston RE. HEB, a helix-loop-helix protein related to E2A and ITF2 that can modulate the DNA-binding ability of myogenic regulatory factors. Mol Cell Biol (1992) 12(3):1031–42. doi: 10.1128/mcb.12.3.1031-1042.1992 PMC369535 1312219

[5] Henthorn P Kiledjian M Kadesch T. Two distinct transcription factors that bind the immunoglobin enhancer mE5/kE2 motif. Sci (1990) 247:467–70. doi: 10.1126/science.2105528 2105528

[6] Aronheim A Shiran R Rosen A Walker MD. The E2A gene product contains two separable and functionally distinct transcription activation domains. Proc Natl Acad Sci USA (1993) 90:8063–7. doi: 10.1073/pnas.90.17.8063 PMC47288 8367464

[7] Massari ME Grant PA Pray-Grant MG Berger SL Workman JL Murre C. A conserved motif present in a class of helix-loop-helix proteins activates transcription by direct recruitment of the SAGA complex. Mol Cell (1999) 4(1):63–73. doi: 10.1016/S1097-2765(00)80188-4 10445028

[8] Quong MW Massari ME Zwart R Murre C. A new transcriptional-activation motif restricted to a class of helix-loop-helix proteins is functionally conserved in both yeast and mammalian cells. Mol Cell Biol (1993) 13:792–800. doi: 10.1128/mcb.13.2.792-800.1993 8423802 PMC358962

[9] Murre C McCaw PS Vaessin H Caudy M Jan LY Jan YN. Interactions between heterologous helix-loop-helix proteins generate complexes that bind specifically to a common DNA sequence. Cell (1989) 58:537–44. doi: 10.1016/0092-8674(89)90434-0 2503252

[10] Kamps MP Murre C Sun X-H Baltimore D. A new homeobox gene contributes the DNA-binding domain of the t(1:19) translocation protein in pre-b ALL. Cell (1990) 60:547–55. doi: 10.1016/0092-8674(90)90658-2 1967983

[11] Sun X-H Baltimore D. An inhibitory domain of E12 prevents DNA binding in E12 homodimers but not in E12 heterodimers. Cell (1991) 64:459–70. doi: 10.1016/0092-8674(91)90653-G 1846322

[12] Wang D Claus CL Vaccarelli G Braunstein M Schmitt TM Zuniga-Pflucker JC. The basic helix-loop-helix transcription factor HEBAlt is expressed in pro-T cells and enhances the generation of T cell precursors. J Immunol (2006) 177(1):109–19. doi: 10.4049/jimmunol.177.1.109 16785505

[13] Yoganathan K Yan A Rocha J Trotman-Grant A Mohtashami M Wells L. Regulation of the signal-dependent e protein HEBAlt through a YYY motif is required for progression through T cell development. Front Immunol (2022) 13:848577). doi: 10.3389/fimmu.2022.848577 35990644 PMC9385190

[14] Benezra R Davis RL Lockshon D Turner DL Weintraub H. The protein id: A negative regulator of helix-loop-helix DNA binding proteins. Cell (1990) 61:49–59. doi: 10.1016/0092-8674(90)90214-Y 2156629

[15] Sun X-H Copeland NG Jenkins NA Baltimore D. Id proteins, Id1 and Id2, selectively inhibit DNA binding by one class of helix-loop-helix proteins. Mol Cell Biol (1991) 11:5603–11. doi: 10.1128/mcb.11.11.5603-5611.1991 PMC361931 1922066

[16] Sun XH. Multitasking of helix-loop-helix proteins in lymphopoiesis. Adv Immunol (2004) 84:43–77. doi: 10.1016/S0065-2776(04)84002-1 15246250

[17] Kee BL. E and ID proteins branch out. Nat Rev Immunol (2009) 9(3):175–84. doi: 10.1038/nri2507 19240756

[18] Christy BA Sanders LK Lau LF Copeland NG Jenkins NA Nathans D. An id-related helix-loop-helix protein encoded by a growth factor-inducible gene. Proc Natl Acad Sci USA (1991) 88:1815–9. doi: 10.1073/pnas.88.5.1815 PMC51116 2000388

[19] Riechmann V van Cruchten I Sablitzky F. The expression pattern of Id4, a novel dominant negative helix-loop-helix protein, is distinct from Id1, Id2 and Id3. Nucl Acids Res (1994) 22:749–55. doi: 10.1093/nar/22.5.749 PMC307878 8139914

[20] Adolfsson J Borge OJ Bryder D Theilgaard-Monch K Astrand-Grundstrom I Sitnicka E. Upregulation of Flt3 expression within the bone marrow lin(-)Sca1(+)c-kit(+) stem cell compartment is accompanied by loss of self-renewal capacity. Immun (2001) 15(4):659–69. doi: 10.1016/S1074-7613(01)00220-5 11672547

[21] Kondo M Weissman IL Akashi K. Identification of clonogenic common lymphoid progenitors in mouse bone marrow. Cell (1997) 91(5):661–72. doi: 10.1016/S0092-8674(00)80453-5 9393859

[22] Perry SS Welner RS Kouro T Kincade PW Sun XH. Primitive lymphoid progenitors in bone marrow with T lineage reconstituting potential. J Immunol (2006) 177(5):2880–7. doi: 10.4049/jimmunol.177.5.2880 PMC1850233 16920923

[23] Allman D Sambandam A Kim S Miller JP Pagan A Well D. Thymopoiesis independent of common lymphoid progenitors. Nat Immunol (2003) 4(2):168–74. doi: 10.1038/ni878 12514733

[24] Fehling HJ von Boehmer H. Early alpha beta T cell development in the thymus of normal and genetically altered mice. Curr Opin Immunol (1997) 9(2):263–75. doi: 10.1016/S0952-7915(97)80146-X 9099797

[25] Godfrey DI Kennedy J Suda T Zlotnik A. A developmental pathway involving four phenotypically and functionally distinct subsets of CD3-CD4-CD8- triple-negative adult mouse thymocytes defined by CD44 and CD25 expression. J Immunol (1993) 150(10):4244–52. 8387091

[26] Kisielow P von Boehmer H. Development and selection of T cells: facts and puzzles. Adv Immunol (1995) 58:87–209. doi: 10.1016/S0065-2776(08)60620-3 7741032

[27] Porritt HE Rumfelt LL Tabrizifard S Schmitt TM Zuniga-Pflucker JC Petrie HT. Heterogeneity among DN1 prothymocytes reveals multiple progenitors with different capacities to generate T cell and non-T cell lineages. Immun (2004) 20(6):735–45. doi: 10.1016/j.immuni.2004.05.004 15189738

[28] Shortman K Wilson A Egerton M Pearse M Scollay R. Immature CD4- CD8+ murine thymocytes. Cell Immunol (1988) 113(2):462–79. doi: 10.1016/0008-8749(88)90042-1 2965990

[29] MacDonald HR Budd RC Howe RC. A CD3- subset of CD4-8+ thymocytes: A rapidly cycling intermediate in the generation of CD4+8+ cells. Eur J Immunol (1988) 18(4):519–23. doi: 10.1002/eji.1830180405 2966738

[30] Prabhu S Ignatova A Park ST Sun X-H. Regulation of the expression of cyclin-dependent kinase inhibitor p21 by E2A and ID proteins. Mol Cell Biol (1997) 17(10):5888–96. doi: 10.1128/MCB.17.10.5888 PMC232436 9315646

[31] Sambandam A Maillard I Zediak VP Xu L Gerstein RM Aster JC. Notch signaling controls the generation and differentiation of early T lineage progenitors. Nat Immunol (2005) 6(7):663–70. doi: 10.1038/ni1216 15951813

[32] Ciofani M Knowles GC Wiest DL von Boehmer H Zuniga-Pflucker JC. Stage-specific and differential notch dependency at the alphabeta and gammadelta T lineage bifurcation. Immun (2006) 25(1):105–16. doi: 10.1016/j.immuni.2006.05.010 16814577

[33] He YW Nakajima H Leonard WJ Adkins B Malek TR. The common gamma-chain of cytokine receptors regulates intrathymic T cell development at multiple stages. J Immunol (1997) 158(6):2592–9. 9058791

[34] Radtke F Wilson A Stark G Bauer M van Meerwijk J MacDonald HR. Deficient T cell fate specification in mice with an induced inactivation of Notch1. Immun (1999) 10(5):547–58. doi: 10.1016/S1074-7613(00)80054-0 10367900

[35] Haks MC Oosterwegel MA Blom B Spits HM Kruisbeek AM. Cell-fate decisions in early T cell development: Regulation by cytokine receptors and the pre-TCR. Semin Immunol (1999) 11(1):23–37. doi: 10.1006/smim.1998.0153 9950750

[36] Okamura RM Sigvardsson M Galceran J Verbeek S Clevers H Grosschedl R. Redundant regulation of T cell differentiation and TCRalpha gene expression by the transcription factors LEF-1 and TCF-1. Immun (1998) 8(1):11–20. doi: 10.1016/s1074-7613(00)80454-9 9462507

[37] Anderson MK Hernandez-Hoyos G Dionne CJ Arias AM Chen D Rothenberg EV. Definition of regulatory network elements for T cell development by perturbation analysis with PU.1 and GATA-3. Dev Biol (2002) 246(1):103–21. doi: 10.1006/dbio.2002.0674 12027437

[38] Weber BN Chi AW Chavez A Yashiro-Ohtani Y Yang Q Shestova O. A critical role for TCF-1 in T-lineage specification and differentiation. Nat (2011) 476(7358):63–8. doi: 10.1038/nature10279 PMC3156435 21814277

[39] Tydell CC David-Fung ES Moore JE Rowen L Taghon T Rothenberg EV. Molecular dissection of prethymic progenitor entry into the T lymphocyte developmental pathway. J Immunol (2007) 179(1):421–38. doi: 10.4049/jimmunol.179.1.421 17579063

[40] Hosokawa H Romero-Wolf M Yang Q Motomura Y Levanon D Groner Y. Cell type-specific actions of Bcl11b in early T-lineage and group 2 innate lymphoid cells. J Exp Med (2020) 217(1):e20190972. doi: 10.1084/jem.20190972 31653691 PMC7037248

[41] Mallick CA Dudley EC Viney JL Owen MJ Hayday AC. Rearrangement and diversity of T cell receptor beta chain genes in thymocytes: A critical role for the beta chain in development. Cell (1993) 73(3):513–9. doi: 10.1016/0092-8674(93)90138-G 8387894

[42] Hoffman ES Passoni L Crompton T Leu TM Schatz DG Koff A. Productive T-cell receptor beta-chain gene rearrangement: Coincident regulation of cell cycle and clonality during development in vivo. Genes Dev (1996) 10(8):948–62. doi: 10.1101/gad.10.8.948 8608942

[43] Schatz DG Oettinger MA Baltimore D. The V(D)J recombination activating gene, RAG-1. Cell (1989) 59(6):1035–48. doi: 10.1016/0092-8674(89)90760-5 2598259

[44] Oettinger MA Schatz DG Gorka C Baltimore D. RAG-1 and RAG-2, adjacent genes that synergistically activate V(D)J recombination. Sci (1990) 248(4962):1517–23. doi: 10.1126/science.2360047 2360047

[45] von Boehmer H Aifantis I Feinberg J Lechner O Saint-Ruf C Walter U. Pleiotropic changes controlled by the pre-t-cell receptor. Curr Opin Immunol (1999) 11(2):135–42. doi: 10.1016/S0952-7915(99)80024-7 10322152

[46] Born W Yague J Palmer E Kappler J Marrack P. Rearrangement of T-cell receptor beta-chain genes during T-cell development. Proc Natl Acad Sci U S A (1985) 82(9):2925–9. doi: 10.1073/pnas.82.9.2925 PMC397679 3873070

[47] Hayday AC Diamond DJ Tanigawa G Heilig JS Folsom V Saito H. Unusual organization and diversity of T-cell receptor alpha-chain genes. Nat (1985) 316(6031):828–32. doi: 10.1038/316828a0 2993907

[48] Yague J Blackman M Born W Marrack P Kappler J Palmer E. The structure of V alpha and J alpha segments in the mouse. Nucleic Acids Res (1988) 16(23):11355–64. doi: 10.1093/nar/16.23.11355 PMC339015 2849763

[49] von Boehmer H. Positive selection of lymphocytes. Cell (1994) 76(2):219–28. doi: 10.1016/0092-8674(94)90330-1 8293460

[50] McCaughtry TM Wilken MS Hogquist KA. Thymic emigration revisited. J Exp Med (2007) 204(11):2513–20. doi: 10.1084/jem.20070601 PMC2118501 17908937

[51] Matloubian M Lo CG Cinamon G Lesneski MJ Xu Y Brinkmann V. Lymphocyte egress from thymus and peripheral lymphoid organs is dependent on S1P receptor 1. Nat (2004) 427(6972):355–60. doi: 10.1038/nature02284 14737169

[52] Allende ML Dreier JL Mandala S Proia RL. Expression of the sphingosine 1-phosphate receptor, S1P1, on T-cells controls thymic emigration. J Biol Chem (2004) 279(15):15396–401. doi: 10.1074/jbc.M314291200 14732704

[53] Jin R Wang W Yao JY Zhou YB Qian XP Zhang J. Characterization of the *in vivo* dynamics of medullary CD4+CD8- thymocyte development. J Immunol (2008) 180(4):2256–63. doi: 10.4049/jimmunol.180.4.2256 18250433

[54] Singer A Bosselut R. CD4/CD8 coreceptors in thymocyte development, selection, and lineage commitment: Analysis of the CD4/CD8 lineage decision. Adv Immunol (2004) 83:91–131. doi: 10.1016/S0065-2776(04)83003-7 15135629

[55] Murphy KM Reiner SL. The lineage decisions of helper T cells. Nat Rev Immunol (2002) 2(12):933–44. doi: 10.1038/nri954 12461566

[56] Xiong N Raulet DH. Development and selection of gammadelta T cells. Immunol Rev (2007) 215:15–31. doi: 10.1111/j.1600-065X.2006.00478.x 17291276

[57] Munoz-Ruiz M Sumaria N Pennington DJ Silva-Santos B. Thymic determinants of gammadelta T cell differentiation. Trends Immunol (2017) 38(5):336–44. doi: 10.1016/j.it.2017.01.007 28285814

[58] Parker ME Ciofani M. Regulation of gammadelta T cell effector diversification in the thymus. Front Immunol (2020) 11:42. doi: 10.3389/fimmu.2020.00042 32038664 PMC6992645

[59] Kang J Raulet DH. Events that regulate differentiation of alpha beta TCR+ and gamma delta TCR+ T cells from a common precursor. Semin Immunol (1997) 9(3):171–9. doi: 10.1006/smim.1997.0069 9200328

[60] Krangel MS McMurry MT Hernandez-Munain C Zhong XP Carabana J. Accessibility control of T cell receptor gene rearrangement in developing thymocytes. the TCR alpha/delta locus. Immunol Res (2000) 22(2-3):127–35. doi: 10.1385/IR:22:2-3:127 11339350

[61] Krangel MS. Mechanics of T cell receptor gene rearrangement. Curr Opin Immunol (2009) 21(2):133–9. doi: 10.1016/j.coi.2009.03.009 PMC2676214 19362456

[62] Pereira P Zijlstra M McMaster J Loring JM Jaenisch R Tonegawa S. Blockade of transgenic gamma delta T cell development in beta 2-microglobulin deficient mice. EMBO J (1992) 11(1):25–31. doi: 10.1002/j.1460-2075.1992.tb05023.x 1531458 PMC556421

[63] Kreslavsky T Garbe AI Krueger A von Boehmer H. T Cell receptor-instructed alphabeta versus gammadelta lineage commitment revealed by single-cell analysis. J Exp Med (2008) 205(5):1173–86. doi: 10.1084/jem.20072425 PMC2373848 18443226

[64] Ferrick DA Schrenzel MD Mulvania T Hsieh B Ferlin WG Lepper H. Differential production of interferon-gamma and interleukin-4 in response to Th1- and Th2-stimulating pathogens by gamma delta T cells in vivo. Nature (1995) 373(6511):255–7. doi: 10.1038/373255a0 7816142

[65] Jensen KD Su X Shin S Li L Youssef S Yamasaki S. Thymic selection determines gammadelta T cell effector fate: Antigen-naive cells make interleukin-17 and antigen-experienced cells make interferon gamma. Immun (2008) 29(1):90–100. doi: 10.1016/j.immuni.2008.04.022 PMC2601709 18585064

[66] Wencker M Turchinovich G Di Marco Barros R Deban L Jandke A Cope A. Innate-like T cells straddle innate and adaptive immunity by altering antigen-receptor responsiveness. Nat Immunol (2014) 15(1):80–7. doi: 10.1038/ni.2773 PMC6485477 24241693

[67] Hayes SM Li L Love PE. TCR signal strength influences alphabeta/gammadelta lineage fate. Immun (2005) 22(5):583–93. doi: 10.1016/j.immuni.2005.03.014 15894276

[68] Haks MC Lefebvre JM Lauritsen JP Carleton M Rhodes M Miyazaki T. Attenuation of gammadeltaTCR signaling efficiently diverts thymocytes to the alphabeta lineage. Immun (2005) 22(5):595–606. doi: 10.1016/j.immuni.2005.04.003 15894277

[69] Kreslavsky T Savage AK Hobbs R Gounari F Bronson R Pereira P. TCR-inducible PLZF transcription factor required for innate phenotype of a subset of gammadelta T cells with restricted TCR diversity. Proc Natl Acad Sci U S A (2009) 106(30):12453–8. doi: 10.1073/pnas.0903895106 PMC2718370 19617548

[70] Tarakhovsky A Kanner SB Hombach J Ledbetter JA Muller W Killeen N. A role for CD5 in TCR-mediated signal transduction and thymocyte selection. Sci (1995) 269:535–7. doi: 10.1126/science.7542801 7542801

[71] Azzam HS Grinberg A Lui K Shen H Shores EW Love PE. CD5 expression is developmentally regulated by T cell receptor (TCR) signals and TCR avidity. J Exp Med (1998) 188(12):2301–11. doi: 10.1084/jem.188.12.2301 PMC2212429 9858516

[72] Fahl SP Coffey F Kain L Zarin P Dunbrack RL Jr. Teyton L. Role of a selecting ligand in shaping the murine gammadelta-TCR repertoire. Proc Natl Acad Sci U S A (2018) 115(8):1889–94. doi: 10.1073/pnas.1718328115 PMC5828614 29432160

[73] Lauritsen JP Wong GW Lee SY Lefebvre JM Ciofani M Rhodes M. Marked induction of the helix-loop-helix protein Id3 promotes the gammadelta T cell fate and renders their functional maturation notch independent. Immun (2009) 31(4):565–75. doi: 10.1016/j.immuni.2009.07.010 PMC2768560 19833086

[74] Turchinovich G Hayday AC. Skint-1 identifies a common molecular mechanism for the development of interferon-gamma-secreting versus interleukin-17-secreting gammadelta T cells. Immun (2011) 35(1):59–68. doi: 10.1016/j.immuni.2011.04.018 21737317

[75] Yin Z Chen C Szabo SJ Glimcher LH Ray A Craft J. T-Bet expression and failure of GATA-3 cross-regulation lead to default production of IFN-gamma by gammadelta T cells. J Immunol (2002) 168(4):1566–71. doi: 10.4049/jimmunol.168.4.1566 11823483

[76] Sumaria N Grandjean CL Silva-Santos B Pennington DJ. Strong TCRgammadelta signaling prohibits thymic development of IL-17A-Secreting gammadelta T cells. Cell Rep (2017) 19(12):2469–76. doi: 10.1016/j.celrep.2017.05.071 PMC5489697 28636936

[77] Barros-Martins J Schmolka N Fontinha D Pires de Miranda M Simas JP Brok I. Effector gammadelta T cell differentiation relies on master but not auxiliary Th cell transcription factors. J Immunol (2016) 196(9):3642–52. doi: 10.4049/jimmunol.1501921 26994218

[78] Coffey F Lee SY Buus TB Lauritsen JP Wong GW Joachims ML. The TCR ligand-inducible expression of CD73 marks gammadelta lineage commitment and a metastable intermediate in effector specification. J Exp Med (2014) 211(2):329–43. doi: 10.1084/jem.20131540 PMC3920555 24493796

[79] Garman RD Doherty PJ Raulet DH. Diversity, rearrangement, and expression of murine T cell gamma genes. Cell (1986) 45(5):733–42. doi: 10.1016/0092-8674(86)90787-7 3486721

[80] Xiong N Kang C Raulet DH. Positive selection of dendritic epidermal gammadelta T cell precursors in the fetal thymus determines expression of skin-homing receptors. Immun (2004) 21(1):121–31. doi: 10.1016/j.immuni.2004.06.008 15345225

[81] Havran WL Chien YH Allison JP. Recognition of self antigens by skin-derived T cells with invariant gamma delta antigen receptors. Sci (1991) 252(5011):1430–2. doi: 10.1126/science.1828619 1828619

[82] Narayan K Sylvia KE Malhotra N Yin CC Martens G Vallerskog T. Intrathymic programming of effector fates in three molecularly distinct gammadelta T cell subtypes. Nat Immunol (2012) 13(5):511–8. doi: 10.1038/ni.2247 PMC3427768 22473038

[83] Haas JD Ravens S Duber S Sandrock I Oberdorfer L Kashani E. Development of interleukin-17-producing gammadelta T cells is restricted to a functional embryonic wave. Immun (2012) 37(1):48–59. doi: 10.1016/j.immuni.2012.06.003 22770884

[84] Bain G Engel I Robanus Maandag EC te Riele HP Voland JR Sharp LL. E2A deficiency leads to abnormalities in alphabeta T-cell development and to rapid development of T-cell lymphomas. Mol Cell Biol (1997) 17(8):4782–91. doi: 10.1128/MCB.17.8.4782 PMC232330 9234734

[85] Yan W Young AZ Soares VC Kelley R Benezra R Zhuang Y. High incidence of T-cell tumors in E2A-null mice and E2A/Id1 double-knockout mice. Mol Cell Biol (1997) 17:7317–27. doi: 10.1128/MCB.17.12.7317 PMC232588 9372963

[86] Barndt R Dai MF Zhuang Y. A novel role for HEB downstream or parallel to the pre-TCR signaling pathway during alpha beta thymopoiesis. J Immunol (1999) 163(6):3331–43. 10477603

[87] Kim D Peng XC Sun XH. Massive apoptosis of thymocytes in T-cell-deficient Id1 transgenic mice. Mol Cell Biol (1999) 19(12):8240–53. doi: 10.1128/MCB.19.12.8240 PMC84908 10567549

[88] Wang HC Perry SS Sun XH. Id1 attenuates notch signaling and impairs T-cell commitment by elevating Deltex1 expression. Mol Cell Biol (2009) 29(17):4640–52. doi: 10.1128/MCB.00119-09 PMC2725715 19564409

[89] Wojciechowski J Lai A Kondo M Zhuang Y. E2A and HEB are required to block thymocyte proliferation prior to pre-TCR expression. J Immunol (2007) 178(9):5717–26. doi: 10.4049/jimmunol.178.9.5717 PMC2265380 17442955

[90] Miyazaki M Miyazaki K Chen K Jin Y Turner J Moore AJ. The e-id protein axis specifies adaptive lymphoid cell identity and suppresses thymic innate lymphoid cell development. Immun (2017) 46(5):818–34. doi: 10.1016/j.immuni.2017.04.022 PMC5512722 28514688

[91] Yashiro-Ohtani Y He Y Ohtani T Jones ME Shestova O Xu L. Pre-TCR signaling inactivates Notch1 transcription by antagonizing E2A. Genes Dev (2009) 23(14):1665–76. doi: 10.1101/gad.1793709 PMC2714710 19605688

[92] Yuan JS Kousis PC Suliman S Visan I Guidos CJ. Functions of notch signaling in the immune system: consensus and controversies. Annu Rev Immunol (2010) 28:343–65. doi: 10.1146/annurev.immunol.021908.132719 20192807

[93] Hsu LY Lauring J Liang HE Greenbaum S Cado D Zhuang Y. A conserved transcriptional enhancer regulates RAG gene expression in developing b cells. Immun (2003) 19(1):105–17. doi: 10.1016/S1074-7613(03)00181-X 12871643

[94] Miyazaki K Watanabe H Yoshikawa G Chen K Hidaka R Aitani Y. The transcription factor E2A activates multiple enhancers that drive rag expression in developing T and b cells. Sci Immunol (2020) 5(51):eabb1455. doi: 10.1126/sciimmunol.abb1455 32887843

[95] Bain G Romanow WJ Albers K Havran WL Murre C. Positive and negative regulation of V(D)J recombination by the E2A proteins. J Exp Med (1999) 189(2):289–300. doi: 10.1084/jem.189.2.289 9892611 PMC2192990

[96] Barndt RJ Dai M Zhuang Y. Functions of E2A-HEB heterodimers in T-cell development revealed by a dominant negative mutation of HEB. Mol Cell Biol (2000) 20(18):6677–85. doi: 10.1128/MCB.20.18.6677-6685.2000 PMC86175 10958665

[97] Takeuchi A Yamasaki S Takase K Nakatsu F Arase H Onodera M. E2A and HEB activate the pre-TCR alpha promoter during immature T cell development. J Immunol (2001) 167(4):2157–63. doi: 10.4049/jimmunol.167.4.2157 11490000

[98] Tremblay M Herblot S Lecuyer E Hoang T. Regulation of pT alpha gene expression by a dosage of E2A, HEB, and SCL. J Biol Chem (2003) 278(15):12680–7. doi: 10.1074/jbc.M209870200 12566462

[99] Emmanuel AO Arnovitz S Haghi L Mathur PS Mondal S Quandt J. TCF-1 and HEB cooperate to establish the epigenetic and transcription profiles of CD4(+)CD8(+) thymocytes. Nat Immunol (2018) 19(12):1366–78. doi: 10.1038/s41590-018-0254-4 PMC6867931 30420627

[100] Alberola-Ila J Hogquist KA Swan KA Bevan MJ Perlmutter RM. Positive and negative selection invoke distinct signaling pathways. J Exp Med (1996) 184(1):9–18. doi: 10.1084/jem.184.1.9 8691153 PMC2192689

[101] Bain G Cravatt CB Loomans C Alberola-Ila J Hedrick SM Murre C. Regulation of the helix-loop-helix proteins, E2A and Id3, by the ras- ERK MAPK cascade. Nat Immunol (2001) 2(2):165–71. doi: 10.1038/84273 11175815

[102] Bettini M Xi H Milbrandt J Kersh GJ. Thymocyte development in early growth response gene 1-deficient mice. J Immunol (2002) 169(4):1713–20. doi: 10.4049/jimmunol.169.4.1713 12165491

[103] Haks MC Krimpenfort P Borst J Kruisbeek AM. The CD3g chain is essential for development of both the TCRab and TCRgd lineages. EMBO J (1998) 17:1871–82. doi: 10.1093/emboj/17.7.1871 PMC1170534 9524111

[104] Mombaerts P Iacomini J Johnson RS Herrup K Tonegawa S Papaioannou VE. RAG-1 deficient mice have no mature b and T lymphocytes. Cell (1992) 68:869–77. doi: 10.1016/0092-8674(92)90030-G 1547488

[105] Kim D Xu M Nie L Peng XC Jimi E Voll RE. Helix-loop-helix proteins regulate pre-TCR and TCR signaling through modulation of Rel/NF-kappaB activities. Immun (2002) 16(1):9–21. doi: 10.1016/S1074-7613(02)00264-9 11825562

[106] Engel I Johns C Bain G Rivera RR Murre C. Early thymocyte development is regulated by modulation of E2A protein activity. J Exp Med (2001) 194(6):733–45. doi: 10.1084/jem.194.6.733 PMC2195962 11560990

[107] Nie L Xu M Vladimirova A Sun XH. Notch-induced E2A ubiquitination and degradation are controlled by MAP kinase activities. EMBO J (2003) 22(21):5780–92. doi: 10.1093/emboj/cdg567 PMC275424 14592976

[108] Nie L Perry SS Zhao Y Huang J Kincade PW Farrar MA. Regulation of lymphocyte development by cell-type-specific interpretation of notch signals. Mol Cell Biol (2008) 28(6):2078–90. doi: 10.1128/MCB.00844-07 PMC2268404 18195039

[109] Rivera RR Johns CP Quan J Johnson RS Murre C. Thymocyte selection is regulated by the helix-loop-helix inhibitor protein, Id3. Immun (2000) 12:17–26. doi: 10.1016/S1074-7613(00)80155-7 10661402

[110] Jones-Mason ME Zhao X Kappes D Lasorella A Iavarone A Zhuang Y. E protein transcription factors are required for the development of CD4(+) lineage T cells. Immun (2012) 36(3):348–61. doi: 10.1016/j.immuni.2012.02.010 PMC3431168 22425249

[111] Qi Z Sun XH. Hyperresponse to T-cell receptor signaling and apoptosis of Id1 transgenic thymocytes. Mol Cell Biol (2004) 24(17):7313–23. doi: 10.1128/MCB.24.17.7313-7323.2004 PMC506975 15314144

[112] Yang Y Liou HC Sun XH. Id1 potentiates NF-kappaB activation upon T cell receptor signaling. J Biol Chem (2006) 281(46):34989–96. doi: 10.1074/jbc.M608078200 17012234

[113] Ueda-Hayakawa I Mahlios J Zhuang Y. Id3 restricts the developmental potential of gamma delta lineage during thymopoiesis. J Immunol (2009) 182(9):5306–16. doi: 10.4049/jimmunol.0804249 PMC2688458 19380777

[114] Fahl SP Kappes DJ Wiest DL. TCR signaling circuits in alphabeta/gammadelta T lineage choice. In: Soboloff J Kappes DJ, editors. Signaling mechanisms regulating T cell diversity and function. Boca Raton (FL):CRC Press. (2018). p. 85–104.

[115] In TSH Trotman-Grant A Fahl S Chen ELY Zarin P Moore AJ. HEB is required for the specification of fetal IL-17-producing gammadelta T cells. Nat Commun (2017) 8(1):2004. doi: 10.1038/s41467-017-02225-5 29222418 PMC5722817

[116] Yang Y Wang HC Sun XH. Id1 induces apoptosis through inhibition of RORgammat expression. BMC Immunol (2008) 9:20. doi: 10.1186/1471-2172-9-20 18489764 PMC2408562

[117] Spits H Artis D Colonna M Diefenbach A Di Santo JP Eberl G. Innate lymphoid cells–a proposal for uniform nomenclature. Nat Rev Immunol (2013) 13(2):145–9. doi: 10.1038/nri3365 23348417

[118] Eberl G Colonna M Di Santo JP McKenzie AN. Innate lymphoid cells. innate lymphoid cells: a new paradigm in immunology. Science (2015) 348(6237):aaa6566. doi: 10.1126/science.aaa6566 25999512 PMC5658207

[119] Zook EC Kee BL. Development of innate lymphoid cells. Nat Immunol (2016) 17(7):775–82. doi: 10.1038/ni.3481 27328007

[120] Yang Q Bhandoola A. The development of adult innate lymphoid cells. Curr Opin Immunol (2016) 39:114–20. doi: 10.1016/j.coi.2016.01.006 PMC4801723 26871595

[121] Ricardo-Gonzalez RR Van Dyken SJ Schneider C Lee J Nussbaum JC Liang HE. Tissue signals imprint ILC2 identity with anticipatory function. Nat Immunol (2018) 19(10):1093–9. doi: 10.1038/s41590-018-0201-4 PMC6202223 30201992

[122] Vivier E Artis D Colonna M Diefenbach A Di Santo JP Eberl G. Innate lymphoid cells: 10 years on. Cell (2018) 174(5):1054–66. doi: 10.1016/j.cell.2018.07.017 30142344

[123] Artis D Spits H. The biology of innate lymphoid cells. Nat (2015) 517(7534):293–301. doi: 10.1038/nature14189 25592534

[124] Hoyler T Klose CS Souabni A Turqueti-Neves A Pfeifer D Rawlins EL. The transcription factor GATA-3 controls cell fate and maintenance of type 2 innate lymphoid cells. Immun (2012) 37(4):634–48. doi: 10.1016/j.immuni.2012.06.020 PMC3662874 23063333

[125] Yagi R Zhong C Northrup DL Yu F Bouladoux N Spencer S. The transcription factor GATA3 is critical for the development of all IL-7Ralpha-expressing innate lymphoid cells. Immun (2014) 40(3):378–88. doi: 10.1016/j.immuni.2014.01.012 PMC4026797 24631153

[126] Zhu J. GATA3 regulates the development and functions of innate lymphoid cell subsets at multiple stages. Front Immunol (2017) 8:1571. doi: 10.3389/fimmu.2017.01571 29184556 PMC5694433

[127] Wong SH Walker JA Jolin HE Drynan LF Hams E Camelo A. Transcription factor RORalpha is critical for nuocyte development. Nat Immunol (2012) 13(3):229–36. doi: 10.1038/ni.2208 PMC3343633 22267218

[128] Serafini N Vosshenrich CA Di Santo JP. Transcriptional regulation of innate lymphoid cell fate. Nat Rev Immunol (2015) 15(7):415–28. doi: 10.1038/nri3855 26065585

[129] Monticelli LA Sonnenberg GF Abt MC Alenghat T Ziegler CG Doering TA. Innate lymphoid cells promote lung-tissue homeostasis after infection with influenza virus. Nat Immunol (2011) 12(11):1045–54. doi: 10.1038/ni.2131 PMC3320042 21946417

[130] Yokota Y Mansouri A Mori S Sugawara S Adachi S Nishikawa S. Development of peripheral lymphoid organs and natural killer cells depends on the helix-loop-helix inhibitor Id2. Nat (1999) 397:702–6. doi: 10.1038/17812 10067894

[131] Wang HC Qian L Zhao Y Mengarelli J Adrianto I Montgomery CG. Downregulation of e protein activity augments an ILC2 differentiation program in the thymus. J Immunol (2017) 198(8):3149–56. doi: 10.4049/jimmunol.1602009 PMC5404348 28258196

[132] Qian L Bajana S Georgescu C Peng V Wang HC Adrianto I. Suppression of ILC2 differentiation from committed T cell precursors by e protein transcription factors. J Exp Med (2019) 216(4):884–99. doi: 10.1084/jem.20182100 PMC6446881 30898894

[133] Bajana S Pankow A Liu K Michniowska M Urban JF Jr. Chen WR. Correlation between circulating innate lymphoid cell precursors and thymic function. iSci (2022) 25(2):103732. doi: 10.1016/j.isci.2022.103732 PMC8792071 35118353

[134] Yu X Wang Y Deng M Li Y Ruhn KA Zhang CC. The basic leucine zipper transcription factor NFIL3 directs the development of a common innate lymphoid cell precursor. Elife (2014) 3:e04406. doi: 10.7554/eLife.04406 PMC4356142 25310240

[135] Yang Q Li F Harly C Xing S Ye L Xia X. TCF-1 upregulation identifies early innate lymphoid progenitors in the bone marrow. Nat Immunol (2015) 16(10):1044–50. doi: 10.1038/ni.3248 PMC4575643 26280998

[136] Klose CS Flach M Mohle L Rogell L Hoyler T Ebert K. Differentiation of type 1 ILCs from a common progenitor to all helper-like innate lymphoid cell lineages. Cell (2014) 157(2):340–56. doi: 10.1016/j.cell.2014.03.030 24725403

[137] Constantinides MG McDonald BD Verhoef PA Bendelac A. A committed precursor to innate lymphoid cells. Nat (2014) 508(7496):397–401. doi: 10.1038/nature13047 PMC4003507 24509713

[138] Seillet C Mielke LA Amann-Zalcenstein DB Su S Gao J Almeida FF. Deciphering the innate lymphoid cell transcriptional program. Cell Rep (2016) 17(2):436–47. doi: 10.1016/j.celrep.2016.09.025 27705792

[139] Bai L Vienne M Tang L Kerdiles Y Etiennot M Escaliere B. Liver type 1 innate lymphoid cells develop locally *via* an interferon-gamma-dependent loop. Science (2021) 371(6536):aba4177. doi: 10.1126/science.aba4177 33766856

[140] Gasteiger G Fan X Dikiy S Lee SY Rudensky AY. Tissue residency of innate lymphoid cells in lymphoid and nonlymphoid organs. Sci (2015) 350(6263):981–5. doi: 10.1126/science.aac9593 PMC4720139 26472762

[141] Huang Y Mao K Chen X Sun MA Kawabe T Li W. S1P-dependent interorgan trafficking of group 2 innate lymphoid cells supports host defense. Sci (2018) 359(6371):114–9. doi: 10.1126/science.aam5809 PMC6956613 29302015

[142] Zeis P Lian M Fan X Herman JS Hernandez DC Gentek R. *In situ* maturation and tissue adaptation of type 2 innate lymphoid cell progenitors. Immun (2020) 53(4):775–92. doi: 10.1016/j.immuni.2020.09.002 PMC7611573 33002412

[143] Ghaedi M Shen ZY Orangi M Martinez-Gonzalez I Wei L Lu X. Single-cell analysis of RORalpha tracer mouse lung reveals ILC progenitors and effector ILC2 subsets. J Exp Med (2020) 217(3):20182293. doi: 10.1084/jem.20182293 PMC7062532 31816636

[144] Lim AI Li Y Lopez-Lastra S Stadhouders R Paul F Casrouge A. Systemic human ILC precursors provide a substrate for tissue ILC differentiation. Cell (2017) 168(6):1086–100. doi: 10.1016/j.cell.2017.02.021 28283063

[145] Nagasawa M Heesters BA Kradolfer CMA Krabbendam L Martinez-Gonzalez I de Bruijn MJW. KLRG1 and NKp46 discriminate subpopulations of human CD117(+)CRTH2(-) ILCs biased toward ILC2 or ILC3. J Exp Med (2019) 216(8):1762–76. doi: 10.1084/jem.20190490 PMC6683990 31201208

[146] Mjosberg J Spits H. Human innate lymphoid cells. J Allergy Clin Immunol (2016) 138(5):1265–76. doi: 10.1016/j.jaci.2016.09.009 27677386

[147] Cosmi L Liotta F Maggi L Annunziato F. Role of type 2 innate lymphoid cells in allergic diseases. Curr Allergy Asthma Rep (2017) 17(10):66. doi: 10.1007/s11882-017-0735-9 28895020

[148] Ebbo M Crinier A Vely F Vivier E. Innate lymphoid cells: major players in inflammatory diseases. Nat Rev Immunol (2017) 17(11):665–78. doi: 10.1038/nri.2017.86 28804130

[149] Xiong T Turner JE. Innate lymphoid cells in autoimmunity and chronic inflammatory diseases. Semin Immunopathol (2018) 40(4):393–406. doi: 10.1007/s00281-018-0670-4 29568972

[150] Jones R Cosway EJ Willis C White AJ Jenkinson WE Fehling HJ. Dynamic changes in intrathymic ILC populations during murine neonatal development. Eur J Immunol (2018) 48(9):1481–91. doi: 10.1002/eji.201847511 PMC6174991 29851080

[151] Kernfeld EM Genga RMJ Neherin K Magaletta ME Xu P Maehr R. A single-cell transcriptomic atlas of thymus organogenesis resolves cell types and developmental maturation. Immun (2018) 48(6):1258–70. doi: 10.1016/j.immuni.2018.04.015 PMC6013397 29884461

[152] Elsaid R Meunier S Burlen-Defranoux O Soares-da-Silva F Perchet T Iturri L. A wave of bipotent T/ILC-restricted progenitors shapes the embryonic thymus microenvironment in a time-dependent manner. Blood (2021) 137(8):1024–36. doi: 10.1182/blood.2020006779 PMC8065239 33025012

[153] Ferreira ACF Szeto ACH Heycock MWD Clark PA Walker JA Crisp A. RORalpha is a critical checkpoint for T cell and ILC2 commitment in the embryonic thymus. Nat Immunol (2021) 22(2):166–78. doi: 10.1038/s41590-020-00833-w PMC7116838 33432227

[154] Liu C Gong Y Zhang H Yang H Zeng Y Bian Z. Delineating spatiotemporal and hierarchical development of human fetal innate lymphoid cells. Cell Res (2021) 31:1106–22. doi: 10.1038/s41422-021-00529-2 PMC8486758 34239074

[155] De Obaldia ME Bhandoola A. Transcriptional regulation of innate and adaptive lymphocyte lineages. Annu Rev Immunol (2015) 33:607–42. doi: 10.1146/annurev-immunol-032414-112032 25665079

[156] Shin SB Lo BC Ghaedi M Scott RW Li Y Messing M. Abortive gammadeltaTCR rearrangements suggest ILC2s are derived from T-cell precursors. Blood Adv (2020) 4(21):5362–72. doi: 10.1182/bloodadvances.2020002758 PMC7656916 33137203

[157] Shin SB McNagny KM. ILC-you in the thymus: A fresh look at innate lymphoid cell development. Front Immunol (2021) 12:681110. doi: 10.3389/fimmu.2021.681110 34025680 PMC8136430

[158] Vargas CL Poursine-Laurent J Yang L Yokoyama WM. Development of thymic NK cells from double negative 1 thymocyte precursors. Blood (2011) 118(13):3570–8. doi: 10.1182/blood-2011-06-359679 PMC3186333 21821702

[159] Veinotte LL Greenwood CP Mohammadi N Parachoniak CA Takei F. Expression of rearranged TCRgamma genes in natural killer cells suggests a minor thymus-dependent pathway of lineage commitment. Blood (2006) 107(7):2673–9. doi: 10.1182/blood-2005-07-2797 16317098

[160] Vosshenrich CA Garcia-Ojeda ME Samson-Villeger SI Pasqualetto V Enault L Richard-Le Goff O. A thymic pathway of mouse natural killer cell development characterized by expression of GATA-3 and CD127. Nat Immunol (2006) 7(11):1217–24. doi: 10.1038/ni1395 17013389

[161] Rankin L Belz GT. Diverse roles of inhibitor of differentiation 2 in adaptive immunity. Clin Dev Immunol (2011) 2011:281569. doi: 10.1155/2011/281569 21437223 PMC3061294

[162] Barshad G Webb LM Ting HA Oyesola OO Onyekwere OG Lewis JJ. E-protein inhibition in ILC2 development shapes the function of mature ILC2s during allergic airway inflammation. J Immunol (2022) 208(5):1007–20. doi: 10.4049/jimmunol.2100414 PMC8881320 35181641

[163] Berrett H Qian L Roman O Cordova A Simmons A Sun XH. Development of type 2 innate lymphoid cells is selectively inhibited by sustained e protein activity. Immunohorizons (2019) 3(12):593–605. doi: 10.4049/immunohorizons.1900045 31852728 PMC6938226

[164] Heemskerk MH Blom B Oda K Stegmann AP Bakker AQ Weijer K. Inhibition of T cell and promotion of natural killer cell development by the dominant negative helix loop helix factor Id3. J Exp Med (1997) 186:1597–602. doi: 10.1084/jem.186.9.1597 PMC2199115 9348318

[165] Boos MD Yokota Y Eberl G Kee BL. Mature natural killer cell and lymphoid tissue-inducing cell development requires Id2-mediated suppression of e protein activity. J Exp Med (2007) 204(5):1119–30. doi: 10.1084/jem.20061959 PMC2118569 17452521

[166] Schotte R Dontje W Nagasawa M Yasuda Y Bakker AQ Spits H. Synergy between IL-15 and Id2 promotes the expansion of human NK progenitor cells, which can be counteracted by the e protein HEB required to drive T cell development. J Immunol (2010) 184(12):6670–9. doi: 10.4049/jimmunol.0901508 20483740

[167] Delconte RB Shi W Sathe P Ushiki T Seillet C Minnich M. The helix-Loop-Helix protein ID2 governs NK cell fate by tuning their sensitivity to interleukin-15. Immun (2016) 44(1):103–15. doi: 10.1016/j.immuni.2015.12.007 26795246

[168] Peng V Georgescu C Bakowska A Pankow A Qian L Wren JD. E proteins orchestrate dynamic transcriptional cascades implicated in the suppression of the differentiation of group 2 innate lymphoid cells. J Biol Chem (2020) 295(44):14866–77. doi: 10.1074/jbc.RA120.013806 PMC7606671 32817168

[169] Amann JM Nip J Strom DK Lutterbach B Harada H Lenny N. ETO, a target of t (8,21) in acute leukemia, makes distinct contacts with multiple histone deacetylases and binds mSin3A through its oligomerization domain. Mol Cell Biol (2001) 21(19):6470–83. doi: 10.1128/MCB.21.19.6470-6483.2001 PMC99794 11533236

[170] Jin C Li H Murata T Sun K Horikoshi M Chiu R. JDP2, a repressor of AP-1, recruits a histone deacetylase 3 complex to inhibit the retinoic acid-induced differentiation of F9 cells. Mol Cell Biol (2002) 22(13):4815–26. doi: 10.1128/MCB.22.13.4815-4826.2002 PMC133911 12052888

[171] Muto A Hoshino H Madisen L Yanai N Obinata M Karasuyama H. Identification of Bach2 as a b-cell-specific partner for small maf proteins that negatively regulate the immunoglobulin heavy chain gene 3' enhancer. EMBO J (1998) 17(19):5734–43. doi: 10.1093/emboj/17.19.5734 PMC1170901 9755173

[172] Chyla BJ Moreno-Miralles I Steapleton MA Thompson MA Bhaskara S Engel M. Deletion of Mtg16, a target of t(16;21), alters hematopoietic progenitor cell proliferation and lineage allocation. Mol Cell Biol (2008) 28(20):6234–47. doi: 10.1128/MCB.00404-08 PMC2577421 18710942

[173] Miller MM Patel PS Bao K Danhorn T O'Connor BP Reinhardt RL. BATF acts as an essential regulator of IL-25-responsive migratory ILC2 cell fate and function. Sci Immunol (2020) 5(43):aay3994. doi: 10.1126/sciimmunol.aay3994 PMC7112987 31924686

[174] Xu W Carr T Ramirez K McGregor S Sigvardsson M Kee BL. E2A transcription factors limit expression of Gata3 to facilitate T lymphocyte lineage commitment. Blood (2013) 121(9):1534–42. doi: 10.1182/blood-2012-08-449447 PMC3587319 23297135

[175] Cherrier DE Serafini N Di Santo JP. Innate lymphoid cell development: A T cell perspective. Immun (2018) 48(6):1091–103. doi: 10.1016/j.immuni.2018.05.010 29924975

